# Sport Education and Sportsmanship Orientations: An Intervention in High School Students

**DOI:** 10.3390/ijerph17030837

**Published:** 2020-01-29

**Authors:** Rafael Burgueño, Jesús Medina-Casaubón

**Affiliations:** 1 Health Research Center, University of Almeria, 04009 Almería, Spain; rbm288@ual.es; 2 Department of Physical Education and Sport, University of Granada, 18071 Granada, Spain

**Keywords:** moral development, ethical development, fair play, sporting behavior, instructional models, models-based practice, skill-drill-game approaches, curriculum and instruction

## Abstract

One of the main goals for physical education is to develop the students’ moral and ethical domain, where sportsmanship promotion is considered a key curricular component to tackle the achievement of this goal. This research aims to examine the influence of sport education on sportsmanship orientations in high school students. The participants were 148 (52.70% female; *M_age_* = 17.04, *SD_age_* = 0.99) high school students who were randomized into an experimental group (*n* = 74), which received 16 basketball lessons under sport education conditions, and a control group (*n* = 74), which received 16 basketball lessons following a traditional teaching approach. Pre-intervention and post-intervention measures on sportsmanship orientations were collected in both groups. A 2 (time: pre-test and post-test) x 2 (group: Sport Education and Traditional Teaching) multivariate analysis of variance test was performed on the five sportsmanship orientations. The results showed, for time x group interactions, the absence of significant multivariate effects in the level of the five sportsmanship orientations among both groups at pre-test (Pillai’s trace = 0.06, *p* = 0.145). At post-test, significant multivariate effects were found in the level of each sportsmanship orientation between both groups in favor of the Sport Education group (Pillai’s trace = 0.38, *p* < 0.001). Furthermore, regarding within-group pre-test to post-test differences, while there were nonsignificant multivariate effects (Pillai’s trace = 0.03, *p* = 0.469) for the Traditional Teaching group; significant multivariate effects (Pillai’s trace = 0.43, *p* < 0.001) were found for the Sport Education group, showing an increase in the level of respect for social conventions, respect for rules and referees, and full commitment and respect for opponents. There were also nonsignificant effects across gender (inter-group analysis: Pillai’s trace = 0.08, *p* = 0.068; time x gender interaction: Pillai’s trace = 0.03, *p* = 0.497) and after-school sports (inter-group analysis: Pillai’s trace = 0.02, *p* = 0.776; time x after-school interaction: Pillai’s trace = 0.01, *p* = 0.981). In conclusion, sport education is an effective pedagogical model to be taken into consideration by physical education teachers in order to optimally promote the high school student’s moral and ethical education via the development of sportsmanship orientations in the context of school physical education.

## 1. Introduction

One of the main goals for high and middle secondary schools is to strengthen human rights as common values of a plural society, and, consequently, to prepare students for the effective exercise of citizenship in a democratic society [1]. On the basis of this, physical education (PE), due to its unique features with respect to interpersonal and social interactions among students in an open space, provides an optimal context for the development of these values [2]. To accomplish this goal, PE focuses on the development of the students’ ethical and moral dimensions in general [3], which has been progressively specified in the display of sporting behaviors [4] measured through different indicators such as sportsmanship [5,6,7].

One of the most commonly used theories to analyze sportsmanship is the social and psychological view of sportsmanship outlined by Vallerand and colleagues [8,9]. According to this social and psychological perspective, sportsmanship is operationalized along five dimensions: (a) respect for social conventions, such as shaking hands with opponents after a match, acknowledgment of opponents’ good performance, as well as being a good winner and loser; (b) respect for rules and referees, reflecting the player’s concern and interest for complying with sport rules and obeying the decisions adopted by referees, even when they are not shared; (c) full commitment to one’s sport, expressing the player’s level in terms of effort, involvement, acknowledgment of failures and attempt to improve his/her skills; (d) respect for opponents, reflecting interest, concern and consideration toward the rival; (e) negative approach to sport, expressing the player’s disruptive behaviors after making a mistake, as well as his/her participation conditioned by individual awards and trophies [9]. Consistent with the theoretical tenets proposed by Vallerand et al. [8], it is considered that players would generally behave in accord with their relative support for these five dimensions of sportsmanship, internalized through social interactions from the environment (i.e., coaches, peers, family, or PE teachers).

Several interventions in PE, aiming to enhance sportsmanship by means of traditional multi-activity units, have been promising as they suggested the possibility of improving the level of sportsmanship displayed by students. In particular, previous works reported an increase in the level of sportsmanship through the incorporation of fair-play dilemmas [10], discussion groups [11], or problem-solving activities [12]. Previous studies also showed to be effective when providing choice in instructional activities, using reproductive teaching styles, and requiring students to engage in cooperative activities [13].

At this point, sportsmanship promotion for students has currently become a predominant component for any national PE curriculum [14]. This consideration represents a challenge for PE teachers in the implementation of pedagogical models that can provide a response to fulfill this curricular demand [3]. One of the most extensively used model in the context of the school PE is sport education (SE) [15,16]. This models-based practice was designed to provide authentic, educationally-rich sports experiences for boys and girls in the context of the school PE [16], which makes it aligned with those more student-centered pedagogical models framed within the current zeitgeist of curriculum and instruction theory [17].

Siedentop, Hastie, and van der Mars [16] listed six non-negotiable features (i.e., seasons, affiliation, regular competition, culminating event, record keeping, and festivity) for SE. Particularly, students are permanently assigned to a team throughout a whole season for learning of a specific sport or curricular content. Teams compete during the season that begins with skills practice and team small-sided games and progresses by means of a series of formal competitions. The season finishes with a culminating event that is used to celebrate both individual and team achievements. As part of one team, each student performs a specific role associated with a series of responsibilities, and the team success will depend on the capacity of students to take the steps necessary to fulfill their roles, which can be in turn completed through “duty role” during the regular competition phase. Finally, SE offers students a festive experience during the culminating event and the end of the season awards celebration.

Through the implementation of these features, Siedentop [15] proposes that a student becomes a competent, literate, and enthusiastic sportsperson. This is to say, the student becomes an expert and competent player who understands and values sports and can distinguish between good and bad sportive practices. Consequently, students would participate and behave in a such way so as to converse, protect, and enhance sports cultures. Further, Siedentop [15] (p. 411) points that “SE should be understood as a process by which sport cultures might grow and prosper as humanizing influences for the lives of citizens”.

Previous research on SE has widely shown its instructional benefits and positive educational implications for students in the context of the school PE [18,19,20,21,22]. Regarding works that have examined the influence of SE on sportsmanship in students, Hastie and Sharpe [23] discovered that the use of a fair play accounting system could develop responsibility and positive social behaviors in elementary school students. In a similar vein, Vidoni and Ward [24] informed that a SE season centered on sportsmanship improved active participation and diminished both the waiting time in instructional activities and off-task behaviors among middle school students. Furthermore, there was a slight enhancement in the number of on-task behaviors between the beginning and the end of the season. Likewise, Perlman and Karp [25] reported an increase of middle school students’ level of autonomy, competence, and self-determined motivation after a SE season focused jointly on sportsmanship and inclusion.

On the other hand, both Ang and Penney [26] and Calderon, Martinez de Ojeda, Valverde, and Méndez-Giménez [27] indicated that a SE season enhanced sportsmanship displayed by elementary school students in general terms. In this same vein, Clarke and Quill [28] underlined that middle school students learnt the rules and acceptable codes of behavior for competition such as the recognition of success and the acceptance of defeat. Similarly, Sinelnikov and Hastie [29] discovered that a SE season evidently enhanced sportsmanship behaviors, although there was a certain confusion with its meaning for middle school students. More specifically, Brock and Hastie [30] reported that elementary school students’ conception of sportsmanship changed as the season progressed. Specifically, students initially described sportsmanship as being polite, not arguing with referees, opponents or members of their own team, and equal participation among all members of the team. Nonetheless, when wining became more important as the season progressed, the students perceived it as the lower-skilled students receiving less game time in favor of those students perceived as higher-skilled. Furthermore, Wahl, Alexander, Sinelnikov, and Cuertner-Smith [31] showed that middle school students developed a stronger sense of sportsmanship after several consecutive SE seasons. Instead, García-López, Gutiérrez, González-Víllora, and Valero-Valenzuela [32] observed that a SE season reduced the number of antisocial and off-task behaviors in elementary school students. On the other hand, the work by Méndez-Giménez, Fernández-Rio and Méndez-Alonso [33] showed that a SE season, regardless of the use of self-made or conventional material, significantly improved the middle school students’ level of respect for social conventions, respect for rules and referees, and respect for opponents in middle school students. Despite that this work was based on the social and psychological view for sportsmanship proposed by Vallerand et al. [8,9], it did not take into account the measure of two of the five orientations (i.e., full commitment and negative approach) that conceptualize sportsmanship. Therefore, its analysis continues to be uncompleted.

In addition, it should be highlighted that the absence of a widespread agreement regarding the conceptualization of sportsmanship [6,34] has likely hampered a solid understanding of how the curricular scaffold of SE could influence the development of students’ sportsmanship in the context of the school PE. Indeed, most of the studies on SE have considered the use of unclear theoretical frameworks for the analysis of sportsmanship. This fact does not allow one to draw solid conclusions about the real impact of the curricular scaffold of SE on the development of sportsmanship given that its conceptualization does not only raised problems of understanding for students but it also varied from one research to another, making it impossible its comparison. On the other hand, to date, there has been no evidence from studies on the influence of SE on sportsmanship among high school students. Thus, it should be considered that the students of this educational stage are characterized by a high level of maturity, abstract thinking, responsibility or autonomy, such that their instructional needs are different from those required by students of previous educational stages [35]. Because of these differences, SE is thought to may have a distinct effect in high school students since the features defining this pedagogical model seem to fit best the instructional needs of this type of student.

Therefore, the objective of this research is to examine the influence of a SE intervention program on the five sportsmanship orientations proposed by Vallerand et al. [8,9] in a sample of high school students in PE. Following the results found in previous studies [26,28,30,33], we hypothesized that a SE intervention would significantly improve the level of social conventions, respect for rules and referees, full commitment and respect for opponents, and it would significantly diminish the level of negative approach in high school students. Moreover, we also hypothesize that a traditional teaching (TT) intervention would keep the same level of each one of the five sportsmanship orientations among high school students in PE.

## 2. Materials and Methods

### 2.1. Participants and Setting

The participants were 148 high school students (70 boys and 78 girls) aged between 16 and 18 years (*M_age_* = 17.04, *SD_age_* = 0.99) from six different PE classrooms in three public high secondary schools located in a city in south-eastern Spain. Regarding their educational background, all students tackled basketball as curricular content in previous academic years through skill-drill-game approaches (also known as traditional teaching, TT); however, none of them was previously instructed under SE conditions. With respect to extracurricular sport, 99 students (50 boys and 49 girls) claimed to practice after-school sport, with a weekly frequency ranging from 1 to 6 days (*M_frequency_* = 3.53, *SD_frequency_* = 1.28). Concerning their ethnic background, 16 (10.81%) students self-reported belonging to ethnic minority communities.

In addition, six (four male and two female) PE in-service teachers took part in this study. All of them claimed to have obtained Bachelors of Science in PE and Sports together with Professional Masters of Education (post-primary PE). Additionally, they self-reported a teaching experience between 5 and 16 years (*M_experience_* = 11.98, *SD_experience_* = 3.99) and, more specifically, they claimed to have implemented both skill-drill-game approaches and SE. In this sense, all of them self-reported experience with SE of at least two academic years. The three public schools were selected in accordance with their previous collaboration with the research team in prior studies. These three schools share similar social, cultural, and economic contexts and are in the same urban area.

### 2.2. Design and Procedure

Following previous research on SE [36,37,38,39], this study adopted a clustered randomized approach with, a priori, a non-equivalent control group and with pre-intervention and post-intervention measures. Due to each one of the three participating schools having already organized all their high school students into two classrooms, it was impossible to randomize them in accordance with an independent variable (pedagogical models). Thus, the two participating classrooms from each school were randomized depending on the two pedagogical models considered in this study, such that a classroom was randomly selected as an experimental or SE group, while the second classroom was consequently assigned as a control or TT group. In this regard, a total of 74 high school students formed the experimental or SE group, while another 74 high school students comprised the control or TT group.

On the other hand, the data collection process was conducted at the beginning and end of the intervention program through a questionnaire measuring the current perception of sportsmanship orientations displayed by the high school students in each time. The questionnaire administration was carried out by the researchers, who explained to the students that their participation was voluntary and anonymous, in addition to emphasizing the absence of valid or incorrect responses given that only their opinion was to be known. Furthermore, the research team was available for the survey respondents to resolve any type of doubt raised during the data collection process. The administration of the questionnaire took place in a classroom environment with an approximate time of 15 mins.

This research was approved by the Ethics Committee on Human Research of the corresponding University (322/CEIH/2017). In addition, the permissions of the three high secondary schools participating and informed consent from the student’s parents or legal guardians are available.

### 2.3. Intervention Program

Prior to the beginning of the intervention program, the research team held three different meetings with the three PE teachers responsible for the intervention under SE conditions in order to establish the objectives, contents, and instructional activities to be taught in each lesson. This same procedure was also followed for the three PE teachers in charge of the intervention under TT conditions. The six PE teachers unanimously agreed to select basketball as the sport to be taught.

#### 2.3.1. Sport Education Program

The basketball SE unit included sixteen 60-min lessons, twice per week for a period of eight weeks in the regular PE schedule. The SE season was composed of three main phases. Specifically, the initial phase was formed by an introductory lesson and a teacher-directed practice sub-phase. In the initial lesson, the teacher introduced the key features of SE, while a student committee, chosen by the students and under the supervision of the teacher, created the teams. It was agreed that each team would be composed of a balanced number of lower-, average- and higher-skilled players, a similar number of boys and girls, and expert basketball players. Once the teams were built, the students also designated the different roles established (main coach, physical trainer, material manager, sports analyst and referee) to each member of their team, in addition to choosing the color of clothes, shield and slogan as distinctive signs of each of the distinct teams created. The teacher-directed practice sub-phase, the second and third lessons, aimed to familiarize the students with the curricular scaffolding structure proposed for this pedagogical model and to develop technical skills and tactical awareness of basketball.

The autonomous practice phase included the fourth to twelfth lessons and was directed by the students. This phase aimed to develop the technical and tactical skills for which team practice and competition were combined. The lesson was structured into (a) a 10-min warm-up led by the physical trainer of each team; (b) a 40-min main-part lead by the main coach, in which modified and small-sided games and a preseason tournament were conducted; and (c) a 5-min cool-down, focused on the reflections of the group related to fair-play dilemmas, responsibility, and team goals, and stretching exercises. Furthermore, the “duty role”, along with the fair-play accounting system, was started during the preseason tournament and was maintained until the end of the season. Regarding the fair-play accounting system, all the teams began the preseason tournament with 10 play-fair points. They could score two points for winning, one for defeat and five fair-play points for each fault-free competition. However, they could lose one point every time a determined team member violated the game rules and three points each time the fair-play rules were broken. These rules of fair play were agreed between the teacher and students at the beginning of this phase. On the other hand, the teacher adopted a role of guide in this phase, more specifically, he/she met the coaches just before the beginning of each lesson in order to tackle the problems found in the prior lesson, explaining briefly the lesson to be taught and supporting his/her instruction and leadership. In addition, the teacher also moved around on the basketball courts, providing each team with general feedback and informing the coach on mistakes for him/her to provide his/her team with specific feedback.

The final phase included a regular competition sub-phase and a culminating event. In the regular competition sub-phase—the 13th, 14th, and 15th lessons—a formal tournament among all the teams was developed. For this end, the lesson was structured into (a) a 10-min warm-up led by the physical trainer; (b) a 30-min main part, in which the different competitions were played; (c) a 10-min phase for the elaboration of reports; and (d) a 5-min cool-down. In the latest lesson of the season, a culminating event took place to decide the ranking of the teams participating and an awards ceremony was held to reward all efforts and accomplishments made by the students, where the teacher was the master of ceremony.

#### 2.3.2. Traditional Teaching Program

The basketball unit was implemented under a skill-drill-game approach format, consisting of sixteen 60-min lessons, twice per week over a period of eight weeks in regular PE schedule. The first 12 lessons focused specifically on basic technical basketball skills and its basic tactical elements. These lessons were organized into (a) a 10-min warm-up; (b) a 40-min main part, consisting of a first phase with tasks centered mainly on the development of basic technical skills, a second phase centered on modified and small-sided games among teams, and a third phase centered on competitions; and (c) a 5-min cool-down, through stretching exercises. The latest four lessons focused on competition, where the teacher randomly formed the different teams for each of the four lessons, refereed the competitions, and moved around on the basket courts in order to check the degree of compliance with the game rules. Throughout all the lessons, the teacher controlled for instructional interactions, activity presentation and structure, time management, and feedback.

#### 2.3.3. Model Fidelity

As the PE teachers had manifested their previous experience with both pedagogical models, a brief 10-hour training course was carried out to emphasize the key features defining SE and TT, respectively. This course relied on the guidelines outlined by Sinelnikov [40] and Calderon and Martínez de Ojeda [41]. In addition to this training course, the three PE teachers responsible for SE were monitored by a researcher with wide expertise in this models-based practice, while the other three PE teachers in charge of TT were monitored by a second researcher with broad expertise in this pedagogical model. Particularly, this monitoring included three action units: (a) analysis lesson-per-lesson throughout the intervention program, (b) meetings just after each lesson to solve problems and doubts, and (c) an external assessment conducted by a single observer for each group [39,42,43]. In particular, the observation record sheet elaborated by Sinelnikov [40], and adapted by Calderon, Martinez de Ojeda and Hastie [43] to the Spanish context, was used to verify the correct implementation of SE in the experimental group, while the observation record sheet developed by Cuevas, García-López and Serra-Olivares [39] in the Spanish context was used to confirm the adequate implementation of TT in the control group. Both assessors also ensured the absence of mismatches between the planned and implemented content in the two instructional conditions.

### 2.4. Measurements

#### Sportsmanship Orientations

The Spanish version [44] of the Multidimensional Sportsmanship Orientations Scale [9] was used to measure sportsmanship orientations in PE. This instrument consists of 25 items grouped into 5 items per factor to assess respect for social conventions (e.g., “Always shake hands after the game”), respect for rules and referees (e.g., “Do not criticize the referee for mistakes against self”), full commitment (e.g., “Maximum effort in practices and games”), respect for opponents (e.g., “Refuse to take an advantage of an injured opponent”) and negative approach (e.g., “Ridicule a less competent opponent”). Each item is measured on a 5-point Likert-type scale, from 1 (does not correspond at all to me) to 5 (corresponds exactly to me).

### 2.5. Data Analysis

The Statistical Package for Social Sciences (IBM SPSS Statistics for Mac, version 25.0; Armonk, NY, USA) was used to analyze statistical data. Table 1 shows absolute and standardized values below 1.96 for the skewness and kurtosis coefficients [45], suggesting that the assumption of normality cannot be rejected. Descriptive statistics (mean and standard deviation) were calculated for each dependent variable. Additionally, descriptive statistics and the percentage of agreement, neutrality and disagreement were also calculated for each of the 25 items. For this estimation, we considered the points four and five as representative of agreement, the point three as a neutral value, while the points one and two as indicative of disagreement. The Cronbach’s alpha coefficient (α), which is acceptable with values over 0.70 [46], was estimated to inspect the reliability of all dependent variables. A 2 (time: pre-test and post-test) x 2 (group: SE and TT) multivariate analysis of variance (MANOVA) test was performed to examine the possible effect of the two instructional conditions (SE and TT) on each dependent variable. In this analysis, gender and after-school sports were introduced as covariates. Prior to 2 x 2 MANOVA, there was the need to analyze the homogeneity of covariances through Box’s test [45]. The results (Box’s *M* = 124.15, *F* [55, 68835.98] = 2.09, *p* < 0.001) indicated the violation of the assumption of homogeneity of covariances, suggesting the use of Pillai’s trace as a test statistic for the multivariate analysis [45]. Effect size was also calculated in terms of partial eta squared. According to the criterion proposed by Field [45], a small effect size is considered to be with values as high as 0.10, medium with values close to 0.25, and large with values equal to 0.50 or higher. The level of statistical significance was set at *p* ≤ 0.05.

## 3. Results

Table 2 shows Cronbach’s alpha values above 0.70 for all the dependent variables considered, except for the negative approach dimension [46]. Overall, these findings implied an adequate level of reliability for each sportsmanship orientation. Table 2 also displays differences in the mean score for all the dependent variables between pre-test and post-test for both instructional conditions. More particularly, Table 3 shows mean scores along with the percentage of agreement, neutrality, and disagreement perceived by the students for the total of items for the SE and TT groups at pre-test and post-test.

A 2 x 2 MANOVA test was performed to examine the effects of time (pre-test and post-test) and group (SE and TT) on each one of the five sportsmanship orientations. Nonsignificant multivariate effects were found either for the gender covariate (inter-group analysis: Pillai’s trace = 0.08, *F* (5.00, 140.00) = 2.25, *p* = 0.068, *η*^2^*_p_* = 0.07; time x gender interaction: Pillai’s trace = 0.03, *F* (5.00, 140.00) = 0.88, *p* = 0.497, *η*^2^*_p_*= 0.03) or the after-school sport covariate (inter-group analysis: Pillai’s trace = 0.02, *F* (5.00, 140.00) = 0.50, *p* = 0.776, *η*^2^*_p_* = 0.02; time x after-school interaction: Pillai’s trace = 0.01, *F* (5.00, 140.00) = 0.14, *p* = 0.981, *η*^2^*_p_* = 0.01).

With respect to time x group interactions, Table 4 shows nonsignificant multivariate effects in the level of the five sportsmanship orientations among both groups at pre-test (Pillai’s trace = 0.06, *F* (5.00, 140.00) = 1.67, *p* = 0.145, *η*^2^_p_ = 0.06), indicating the homogeneity of all dependent variables at the beginning of the intervention program between the SE and TT groups. At post-test, significant multivariate effects (Pillai’s trace = 0.38, *F* (5.00, 140.00) = 16.92, *p* < 0.001, *η*^2^*_p_* = 0.38) were found in the level of the five sportsmanship orientations among the two groups. There was a statistically significant increase in the level of respect for social conventions, respect for rules and referees, full commitment, and respect for opponents.

In relation to within-group pre-test to post-test differences, Table 5 displays a nonsignificant multivariate effect for the TT group (Pillai’s trace = 0.03, *F* (5.00, 140.00) = 0.92, *p* = 0.469, *η*^2^_p_ = 0.03) in the level of the five dependent variables between pre-test and post-test. Instead, a significant multivariate effect was found for the SE group (Pillai’s trace = 0.43, *F* (5.00, 140.00) = 21.10, *p* < 0.001, *η*^2^_p_ = 0.43) between pre-test and post-test. Specifically, there was a statistically significant increase in the level of respect for social conventions, respect for rules and referees, full commitment, and respect for opponents.

## 4. Discussion

The objective of this research was to analyze the influence of SE on the five sportsmanship orientations outlined by Vallerand et al. [8,9] in a sample of high school students during their sports teaching and learning process in the context of school PE. The results revealed that a SE season significantly improved the level of respect for social conventions, respect for rules and referees, full commitment, and respect for opponents, but not the level of negative approach displayed by high school physical education students.

The results emerging from this work showed that a SE season significantly increased the level of respect for social conventions among high school students, supporting one of the hypotheses raised for this study. These findings are also consistent with the results obtained by Méndez-Giménez et al. [33], who reported an increase in middle school students’ perception of respect for social conventions after a SE season, regardless of the use self-made or conventional materials. Furthermore, these results are also in line with those found by Brock and Hastie [30] to the extent that they discovered that middle school students were polite by displaying behaviors such as shaking hands with opponents and acknowledging opponents’ good performance.

A second finding from this work revealed a significant enhancement in the level of respect for rules and referees after a SE season in high school students. On the one hand, these results are in contrast to the findings obtained by Brock and Hastie [30] and Wahl-Alexander et al. [31]. Both studies discovered certain conflicts with respect to the compliance with the rules by determined teams whose members did not exemplify fair-play behaviors given that they attributed a higher relevance to winning as the season progressed. In this regard, Wahl-Alexander et al. [31] emphasized that the agreed nature in the setting of the rules of the game between teacher and students could likely have propitiated some type of breach by determined students with a higher social status. On the other hand, the findings of this research are aligned with those obtained by Méndez-Giménez et al. [33] in the direction that they also reported an improvement in respect for rules after a SE season.

In relation to respect for referees, the results of the present study differed from those reported by Wahl-Alexander et al. [31] in the sense that they highlighted the existence of discrepancies with referees for their dishonest decisions adopted during competitions by middle school students. Conversely, the findings emerging from the current study are delineated with those obtained in previous research [29,30]. Specifically, Brock and Hastie [30], together with Sinelnikov and Hastie [29], stated that middle school students were polite in accepting the referee’s decisions throughout the SE season. These results referred to the increase in respect for referees would be explained by each student having adopted the role of referee over the course of the SE season. Moreover, previous research pointed out that there were positive responses by students in officiating tasks, as well as higher engagement towards this role [47], given that students were worried about the serious and diligent performance of this role in order to avoid conferring advantages to a specific team [29].

A third result derived from this research reflected that a SE season significantly enhanced full commitment in high school students, which underpinned one of the hypotheses posed for this work. These findings are in line with those obtained by Calderon, Martínez de Ojeda, and Hastie [48] and Layne and Hastie [17]. A possible justification would be supported by students during the SE season having the opportunity to experience a higher level of autonomy in their teaching and learning process, fulfilling one of the main instructional needs demanded by students for the educational stage of high school [35]. In this regard, each group of students had the possibility to create their own warm-up session and to develop the instructional activities at their own pace, which probably led to an increase in students’ intrinsic desire to actively participate in PE class [39,42].

In this same vein, autonomy-supporting environments characterizing SE [49,50] facilitate students taking the initiative to engage actively in the different activities developed during the season and, in turn, displaying their ability to successfully complete each task raised. In addition to the increase in the amount of student commitment [17,48], previous research has also indicated gains in the quality of this commitment [42]. In other words, the student’s commitment evolved from a commitment initially based on external contingencies (e.g., to achieve the approval of all members of the team) to one based on the identification of the benefits associated with this pedagogical model (e.g., cooperation, workgroup, or autonomy) [42].

The fourth finding of this study showed a significant gain in the high school students’ level of respect for opponents, supporting one of the hypotheses of the present work. The results are consistent with the results obtained by Méndez-Giménez et al. [33] and Brock and Hastie [30] in the sense that both studies discovered that the students helped other classmates and were friendly with each of them. This fact may be explained by the curricular scaffold of SE promoting a task-involving climate [51] that mainly favors both personal effort and learning and mastery of new skills, contributing to improving the level of cohesion and relationship among students, regardless of whether they are opponents or teammates [38,42]. This would be possible because the success perceived by students in a SE season rests on their personal progress and self-referred evaluation criteria instead of focusing on the comparison of the student’s capacity with the remaining classmates [42,51]. Furthermore, these personal achievements are markedly highlighted in the culminating event by means of the awards ceremony and the festival atmosphere that involves the season under SE conditions [15].

The fifth result did not endorse one of the hypotheses proposed for the present research since there were nonsignificant changes in the level of negative approach after a SE season for high school students. Conversely, there was a slight increase that did not reach the level of statistical significance, making us suggest that the competitive aspects of the SE season could likely have a more marked nuance for certain students, encouraging them to develop determined negative social behaviors [15,32]. Possibly the curricular scaffold of SE partly generated these negative social behaviors, given that the inequality between boys and girls could be accentuated due to boys assuming a higher profile than girls in the most decisive moments of the season through the completion of a greater number of responsibilities and the desire to take the roles perceived as the most important ones [52].

However, it should be noted that the five orientations proposed by Vallerand [8,9] are inter-correlated, as they attempt to conceptualize sportsmanship. In this regard, respect for social conventions, respect for rules and referees, full commitment, and respect for opponents would refer to good sportive practices in the sense that all of them represent behaviors related to shaking hands with opponents after a competition, acknowledging both their good performance and the own failures, displaying interest and concern for the opponents, as well as, complying with the rules and obeying the decisions adopted by referees. In contrast to these good sportive behaviors, there would also be bad sportive practices such as disruptive behaviors after a failure or participation based on obtaining some type of award, reflecting the concept proposed for negative approach. Additionally, it should be emphasized that the relationships among the five sportsmanship orientations are not orthogonal in its nature; this is to say, the increase in the level of a specific sportsmanship orientation (e.g., respect for rules and referees) would not necessarily mean a gain in a second sportsmanship orientation (e.g., respect for social conventions) or a decrease in a third sportsmanship orientation (e.g., negative approach). Thus, the curricular scaffold of SE has fostered the social and interpersonal interactions between teacher and students, promoting the process of internalization of those good sportive practices (i.e., respect for social conventions, respect for rules and referees, full commitment, or respect for opponents) in high school students, making them behave throughout the season in accordance with their relative support for the five sportsmanship orientations [8,9]. Nonetheless, it should be emphasized that the use of a fair play accounting system could imply that the high school students adopted any good sportive practice to serve their own needs. In other words, the students could follow the goal of their compliance not only to “do the right thing” based on moral and ethical principles, but also to do what they had to do to win, which was to obtain a large number of fair play points. In this sense, the role of the fair play accounting system needs to be deeply examined in future studies.

Despite the results obtained, this research has presented a series of limitations that should be considered. Firstly, the use of an intentional sampling method leads us to cautiously interpret the findings, making it necessary for new studies to confirm or discuss the results emerging from this research. Secondly, the external assessment was carried out by a single researcher in each group in accordance with the guidelines proposed in previous studies to ensure the fidelity of both pedagogical models [39,42,43]. Nonetheless, this could compromise the verification of the correct implementation of the key features defining both pedagogical models. In this way, the use of at least two external researchers is recommended to allow one to estimate the degree of agreement between external assessors with respect to the observed actions. Thirdly, the results referring to the negative approach dimension should be circumspectly interpreted due to its marginal Cronbach’s alpha value obtained in this study, which has been previously detected [44,53]. Fourthly, this work considered basketball as the curricular content to be taught, despite that the participating students had already addressed it in previous educational levels. Thus, future studies are needed to use new curricular contents for students in order to examine more accurately the effect of SE on sportsmanship orientations in high school students. Fifthly, this study was developed in the educational stage of high school; therefore, future studies are required to analyze the effect of SE on the five sportsmanship orientations described by Vallerand et al. [8,9] in students from other educational stages (e.g., elementary school, middle school, or higher education). Sixthly, this research has only examined the impact of SE at the beginning and the end of the intervention program, not analyzing the target-dependent variables in each of the three main phases described for SE. Thus, future works should take into account this consideration in order to deeply study in which moment of the season the students internalize each of the five sportsmanship orientations proposed by Vallerand et al. [8,9].

## 5. Conclusions

The results of this research reflect the significant improvement of four of the five sportsmanship orientations (i.e., respect for social conventions, respect for rules and referees, full commitment, and respect for opponents) outlined by Vallerand et al. [8,9] after a SE season. These results would imply that SE may be considered as an optimal pedagogical model to be used by PE teachers in order to fulfill high school curricular demands with respect to sportsmanship promotion as an essential part of student moral and ethical development [14]. Likewise, PE teachers should take into consideration the use of SE as a pedagogical model with the capacity to develop in high school students the skills needed to value sports and discern between good and bad sportive practices through the development of sportsmanship with the ultimate goal to conserve, protect, and enhance sports cultures, and, consequently, to educate students as citizens to take active part in a plural and democratic society [1,14].

## Figures and Tables

**Table 1:** Main features for the implementation of sports education and traditional teaching.

Pedagogical Model	Duration	Student’s Role	Competition Phase	Groups/Team
Sport Education	15 60-min lessons	Active involvement in the decision-making process Autonomous fulfillment of responsibilities	Fair play accounting system Duty role team A formal schedule of competition Record keeping and publicity of results	Created by a selection committee Constant throughout the season Heterogeneous, but balanced teams
Traditional Teaching	15 60-min lessons	Compliance with the instructions provided by the teacher	The teacher refereed the matches and managed the fair play	Created by the teacher Changing throughout the season Heterogeneous teams

**Table 2:** Descriptive statistics and reliability coefficients for the Sport Education and Traditional Teaching Groups.

	Sport Education (*n* = 74)										Traditional Teaching (*n* = 74)									
	Pre-Test					Post-Test					Pre-Test					Post-Test				
	α	*M*	*SD*	γ_1_	γ_2_	α	*M*	*SD*	γ_1_	γ_2_	α	*M*	*SD*	γ_1_	γ_2_	α	*M*	*SD*	γ_1_	γ_2_
R. social conventions	0.79	3.24	0.66	−0.24	−0.55	0.84	4.01	0.72	−0.41	−0.60	0.74	3.05	0.76	−0.25	0.46	0.82	3.10	0.65	−0.24	−0.42
R. rules and referees	0.73	3.66	0.59	−0.47	−0.56	0.77	4.24	0.60	−0.50	−0.38	0.76	3.68	0.51	−0.66	0.58	0.81	3.78	0.54	−0.35	−0.39
Full commitment	0.72	3.28	0.61	−0.69	0.52	0.77	4.14	0.60	−0.14	−1.08	0.76	3.38	0.70	−1.13	1.77	0.78	3.33	064	−0.48	−0.03
R. opponent	0.72	3.35	0.60	−0.09	−0.04	0.72	3.78	0.66	−0.33	−0.30	0.78	3.37	0.67	−0.81	0.92	0.76	3.38	0.73	−1.36	1.40
Negative approach	0.66	2.56	0.68	0.36	−0.29	0.65	2.54	0.84	0.29	−0.78	0.64	2.51	0.52	0.14	−0.55	0.67	2.62	0.67	0.34	−0.70

*Note:* R = Respect for; α = Cronbach’s alpha coefficient; γ_1_ = Standardized skewness coefficient; γ_2_ = Standardized kurtosis coefficient.

**Table 3:** Descriptive statistics for each item according to the Sport Education and Traditional Teaching groups.

	Sport Education (*n* = 74)										Traditional Teaching (*n* = 74)									
	Pre-Test					Post-Test					Pre-Test					Post-Test				
	M	SD	%A	%N	%D	M	SD	%A	%N	%D	M	SD	%A	%N	%D	M	SD	%A	%N	%D
Respect for social conventions																				
Item 1	3.44	0.81	20.30	78.30	1.40	4.05	0.94	74.30	20.30	5.40	2.89	1.07	16.20	44.60	39.20	3.11	1.03	16.20	75.70	8.10
Item 6	3.29	0.91	23.00	71.60	5.40	4.16	0.83	76.30	21.00	2.70	3.01	1.05	48.60	41.90	9.50	3.11	0.91	20.30	75.70	4.10
Item 11	3.07	1.13	20.30	67.50	12.20	4.00	0.74	75.60	23.00	1.40	2.88	0.98	10.80	48.60	40.60	3.03	0.87	32.40	62.20	5.40
Item 16	3.18	1.05	29.70	63.50	6.80	3.91	0.81	71.60	25.70	2.70	3.31	0.82	32.40	63.50	4.10	3.10	0.88	20.30	75.60	4.10
Item 21	3.21	0.97	25.70	68.90	5.40	3.93	0.93	66.20	29.70	4.10	3.16	1.05	6.80	48.70	44.60	3.10	0.93	28.40	67.50	4.10
Respect for rules and referees																				
Item 2	3.75	0.85	17.60	81.00	1.40	4.42	0.70	87.80	12.20	0.00	3.88	0.72	43.30	43.20	13.50	3.93	0.68	37.80	51.40	10.80
Item 7	3.89	0.72	10.80	87.80	1.40	4.43	0.73	91.90	5.40	2.70	3.91	0.67	35.10	55.40	9.50	3.91	0.73	39.50	57.80	2.70
Item 12	3.76	0.75	9.50	87.80	2.70	4.30	0.81	83.80	13.50	2.70	3.68	0.77	12.20	90.50	2.70	3.87	0.71	35.40	61.90	2.70
Item 17	3.03	0.99	36.50	51.30	12.20	3.62	1.19	56.80	27.00	16.20	2.95	0.98	18.90	51.40	29.70	3.13	1.15	31.10	54.00	14.90
Item 22	3.85	0.75	12.20	86.40	1.40	4.39	0.83	90.80	5.40	4.10	3.99	0.69	24.30	64.90	10.80	3.94	0.74	26.80	70.50	2.70
Full commitment																				
Item 3	3.37	0.87	16.20	81.10	2.70	4.27	0.76	86.50	10.80	2.70	3.50	0.91	17.60	77.00	5.40	3.33	0.86	14.90	81.00	4.10
Item 8	3.25	0.93	17.60	78.30	4.10	4.12	0.84	78.30	17.60	4.10	3.31	0.92	21.60	73.00	5.40	3.37	0.85	17.60	78.30	4.10
Item 13	3.22	0.93	18.90	74.30	6.80	4.12	0.75	79.70	18.90	1.40	3.28	0.95	21.60	70.30	8.10	3.37	0.82	14.90	81.00	4.10
Item 18	3.30	0.89	20.30	77.00	2.70	4.13	0.94	74.30	21.60	4.10	3.62	1.05	10.80	82.40	6.80	3.31	1.02	23.00	71.60	5.40
Item 23	3.30	0.76	17.60	81.00	1.40	4.04	0.94	79.70	13.50	6.80	3.20	0.95	21.60	71.60	6.80	3.30	0.93	14.90	79.70	5.40
Respect for opponents																				
Item 4	3.74	0.87	21.60	75.70	2.70	4.20	0.86	79.70	16.20	4.10	3.78	1.06	16.20	75.70	8.10	3.81	0.99	90.50	14.90	5.40
Item 9	3.76	0.84	18.90	78.40	2.70	4.20	0.86	78.30	20.30	1.40	3.94	0.94	12.20	83.70	4.10	3.75	0.91	78.30	17.60	4.10
Item 14	2.98	0.94	48.60	40.60	10.80	3.41	1.08	47.30	36.50	16.20	3.01	1.16	31.10	50.00	18.90	3.09	1.06	46.00	35.10	18.90
Item 19	3.06	1.00	37.80	51.40	10.80	3.64	1.22	60.80	21.60	17.60	3.24	0.98	41.90	51.30	6.80	3.12	1.14	52.70	28.40	18.90
Item 24	3.21	1.13	33.80	52.70	13.50	3.45	1.27	50.00	29.70	20.30	2.90	1.39	24.30	47.30	28.40	3.12	1.34	51.40	27.00	21.60
Negative approach																				
Item 5	3.99	0.87	75.70	21.60	2.70	3.65	0.94	72.90	20.30	6.80	3.95	0.95	64.80	31.10	4.10	4.09	0.86	77.00	20.30	2.70
Item 10	2.03	1.11	12.20	18.90	68.90	2.20	1.34	25.60	20.30	54.10	2.15	1.03	12.20	24.30	63.50	2.24	1.23	20.20	12.20	67.60
Item 15	2.52	1.18	24.40	32.40	43.20	2.42	1.31	29.80	25.70	44.60	2.61	1.23	23.00	29.70	47.30	2.77	1.21	32.40	23.00	44.60
Item 20	2.03	1.16	12.50	17.60	68.90	2.44	1.49	33.80	21.60	44.60	2.07	1.15	12.20	16.20	71.60	2.50	1.32	28.40	13.50	58.10
Item 25	2.21	1.17	16.30	25.70	58.10	2.01	1.29	19.00	20.20	60.80	1.77	0.98	6.70	14.90	78.40	2.24	1.18	17.50	23.00	59.50

*Note*: %A = percentage of agreement; %N = percentage of neutrality; %D = percentage of disagreement.

**Table 4:** Analysis of mean differences between the Sport Education and Traditional Teaching groups (pre-test and post-test) for sportsmanship orientations.

Time	Groups	Variable	*Mdif (SE)*	*p*-Value
		Social conventions	0.17 (0.12)	0.151
		Respect for rules and referees	−0.04 (0.09)	0.703
Pre-test	SE-TT	Full commitment	−0.13 (0.11)	0.220
		Respect for opponents	−0.04 (0.10)	0.674
		Negative approach	0.06 (0.10)	0.578
		Social conventions	0.89 (0.11)	>0.001
		Respect for rules and referees	0.45 (0.09)	>0.001
Post-test	SE-TT	Full commitment	0.81 (0.10)	>0.001
		Respect for opponents	0.39 (0.12)	0.001
		Negative approach	−0.10 (0.13)	0.442

*Note:* SE = sport education; TT = traditional teaching; *Mdif* = mean difference; *SE* = standardized error.

**Table 5:** Analysis within-group pre-test to post-test differences in sportsmanship orientation scores.

Time	Group	Variable	*Mdif (SE)*	*p*-Value
Pre-test to Post-test		Social conventions	0.77 (0.12)	<0.001
		Respect for rules and referees	0.58 (0.10)	<0.001
	Sport Education	Full commitment	0.89 (0.11)	<0.001
		Respect for opponents	0.44 (0.11)	<0.001
		Negative approach	0.04 (0.11)	0.748
		Social conventions	0.04 (0.12)	0.721
		Respect for rules and referees	0.10 (0.10)	0.335
	Traditional Teaching	Full commitment	0.05 (0.11)	0.632
		Respect for opponents	0.01 (0.11)	0.912
		Negative approach	0.12 (0.11)	0.261

*Note*: *Mdif* = mean difference; *SE* = standardized error.
